# Musculoskeletal deformities of Alström syndrome-a review of 55 cases

**DOI:** 10.1186/s13023-025-03867-1

**Published:** 2025-07-17

**Authors:** Subadra Wanninayake, Richard Paisey, Hitesh Dabasia, Ashley Cole, Charlotte Dawson, Tarekegn Hiwot

**Affiliations:** 1 Department of Inherited Metabolic Disorders, University Hospitals Birmingham NHS Foundation Trust, Birmingham, UK; 2 Torbay and South Devon NHS Foundation Trust, Torquay, UK; 3 Milton Keynes University Hospital NHS Foundation Trust, Milton Keynes, UK; 4 Sheffield Children’s NHS Foundation Trust, Sheffield, UK; 5 Institute of Metabolism and Systems Research, University of Birmingham, Birmingham, UK

**Keywords:** Alström syndrome, Scoliosis, Alms1 gene, Skeletal deformity, Scoliosis, Kyphosis, Ciliopathy

## Abstract

**Introduction:**

Alström syndrome (ALMS) is an ultra-rare metabolic disorder caused by biallelic loss-of-function in the *Alms1* gene which encodes a ubiquitously expressed centrosomal protein of the primary cilium. Dual sensory defects, several metabolic and hormonal dysfunctions are frequent in ALMS. Increased musculoskeletal deformities have been observed, though these aspects have not been systematically reviewed. This study characterises the anthropometric, clinical, genetic and imaging features of bone deformities in a large UK cohort with ALMS and describes the details of first documented successful corrective surgery for scoliosis.

**Methods:**

A preliminary study of 13 Alström patients was undertaken to evaluate musculoskeletal deformities. Written consent was obtained after sharing of study information via voice mail or Braille. Questionnaires, clinical examination and radiological evaluations were conducted twice 12 months apart by a rheumatologist and an orthopaedic surgeon. Two patients had scoliosis which required intervention. To discover its prevalence, 42 further AS patients were reviewed. All patients attended the Alstom syndrome UK specialist clinics.

**Results:**

In the detailed survey of 13 patients, all had some degree of musculoskeletal deformities, most commonly partially correctable thoracic kyphosis, brachydactyly, femoral anteversion and pes planus but rarely affecting their daily functioning. In the larger group of 55 patients, 6 had scoliosis requiring intervention; two of whom had spinal deformity requiring surgical correction in adolescence, and one had cervical spine surgery for spondylitis.

**Conclusion:**

ALMS patients tend to have high prevalence of musculoskeletal deformities which may be part of the ciliopathy. Postural adaptation to dual sensory loss resulted in correctable kyphosis, treatable by physiotherapy. Scoliosis requiring intervention is frequent (10.9%), with successful surgery undertaken where indicated.

**Supplementary Information:**

The online version contains supplementary material available at 10.1186/s13023-025-03867-1.

## Introduction

Alström Syndrome (ALMS, OMIM 203800) is an ultra-rare genetic disorder resulting from biallelic loss-of-function mutations in the *Alms1* gene on chromosome 2p13. This gene encodes a centrosomal protein associated with ciliopathies [1–4]. Cilia, which are microtubular projections found on nearly all vertebrate cells, serve as sensory organelles that detect and transduce various extracellular signals, including hormones. The Alms1 protein is predominantly expressed in critical metabolic tissues, including skeletal muscle, bone, adipose tissue, liver, and pancreas.

Beyond the original description of the syndrome as a neuronal hearing loss, early childhood obesity and Type 2 diabetes mellitus (T2DM), the diverse phenotype, including metabolic and endocrine disorders, cardiomyopathy, progressive chronic kidney disease and premature death primarily due to cardiomyopathy and respiratory infection have emerged which can present soon after birth, in childhood or young adult life [4–6]. Extreme insulin resistance (IR) and dyslipidaemia are present from birth. Non-alcoholic fatty liver disease (NAFLD), short stature, hypothyroidism and hypogonadism in males, and polycystic ovarian syndrome (PCOS) in females emerge later in the majority [7–10]. Anecdotal observations of AS patients and the largest review of clinical manifestations [5] have suggested a high prevalence of minor skeletal deformities and kyphosis, but this has not been studied systematically. The frequency and severity of kyphoscoliosis are also not known. The high prevalence of cardiomyopathy involves an additional hazard for the major surgery required to correct kyphoscoliosis in ALMS patients [6, 11, 12].

This study describe firstly detailed examination of skeletal deformities in 13 Alström patients at baseline and after one year. Two cases of this small cohort were found to have scoliosis requiring intervention, so the observational study was enlarged to include all 55 patients attending the Alström syndrome UK national clinic.

## Material and method

### Patients’ involvement

55 patients affected by Alström syndrome, gave informed consent for the DAS study (Defining the phenotype in Alström syndrome-DAS; UKCRN 9044, REC approval—South West ethics approval 10/HO203/33) funded by a UK Science Lottery Grant in association with Alström UK. Patients were recruited from Torbay Hospital and Birmingham Children’s Hospital where NHS funded specialist annual clinic reviews took place. Musculoskeletal deformities were assessed in detail in 13 patients by formal questionnaire, routine clinical examination and skeletal x-ray evaluation by an orthopaedic surgeon and a rheumatologist. After 12 months, the participants were reviewed again with repeated examination and x-ray to confirm main previous findings and assess progression of musculoskeletal deformities. Spinal deformities were characterised by direct observation of the patient when standing and with attempted correction of any abnormal or exaggerated curvature. Anthropometric, clinical, biochemical and imaging findings, and follow up data were collected from clinical notes of all 55 patients in the National Alström Clinic. The 6 patients with scoliosis and one with spinal spondylitis was reviewed in detail. Because of the rarity of Alström syndrome the prevalence of scoliosis was measured in all 55 patients, including the group of 13 originally assessed in detail.

### Statistical analysis

Statistical analyses were conducted using Microsoft Excel. Categorical variables were summarized in terms of number, frequency and percentage.

## Results

Twenty-four out of 55 patients being assessed (43.6%) manifested some degree of kyphosis or scoliosis which was not corrected by postural advice with 6 (10.9%) having thoraco-lumbar scoliosis which required interventions either surgical intervention or brace. Mean [range] age was 27.5 [12–60] years. All had brachydactyly, kyphosis and short stature [mean height (SD) 1.57 (0.9) m]. Anthropometric characteristics and mutation analysis of 24 cases are shown in Table 1 and of the remaining participants on the Table 2. Relevant comorbidities are shown in supplementary Table 1.

**Table 1:** Demographic, genetic and anthropometric characteristics of study participants with either kyphosis and/or scoliosis

Case ID	Sex	Ethnicity	Age (years)	Allele 1 nucleotide change	Allele 2 nucleotide change	Height (m)	Weight (Kg)	BMI (Kg/m^2^)	Deafness	Visual loss
1	Male	WB	36	c.7126dupA	c.7126dupA	1.43	67.7	33.1	Yes	Yes
2	Male	WB	60	c.11875dupt	NK	1.63	75.7	28.4	No	Yes
3	Female	NK	18	ALMS1 exon 13 to 16 deletion	ALMS1 exon 13 to 16 deletion	1.59	101.1	40	Yes	partial
4	Male	WB	28	c.6895delG	c.6895delG	1.58	84.5	34.5	Yes	Yes
5	Male	WB	12	c.10775delC	c.10775delC	1.4	43.05	22	Yes	partial
6	Female	WB	36	c.10775delC	NK	1.45	83.6	39	Yes	Yes
7	Female	SA	13	c.5075delC	c.5075delC	1.48	58.6	26.9	Yes	Yes
8	Male	WB	43	c.10769delC	c.11410C > T	1.61	81.9	34	Yes	Yes
9	Male	WB	42	c.10769delC	c.10477C > T	1.55	55	23	Yes	Yes
10	Male	WB	15	c.7368-7371delAGAT	c.2816 T > A	1.55	66.85	27.7	Yes	Yes
11	Female	WB	29	c.4025_4026delinsA	c.6325G > T	1.58	90.8	36.3	Yes	Yes
12	Female	SA	12	r.7672_10381del	r.7672_10381del	1.36	32.4	17.5	Yes	partial
13	Female	WB	38	NK	NK	1.61	89	35	Yes	Yes
14	Female	WB	29	c.8002C > T	c.10879C > T	1.59	73.5	28	Yes	Yes
15	Female	WB	22	c.6823C > T	c.9535C > T	1.52	64.1	28	Yes	Yes
16	Male	WB	29	c.6299C > A	c.10477C > T	1.6	73.8	28.8	No	Yes
17	Male	WB	23	c.6526C > T	NK	1.66	85.4	30.8	Yes	Yes
18	Male	WB	18	c.8932C > T	c.5356A > G	1.65	88.8	32.9	Yes	Yes
19	Male	WB	15	C.10837C > T	C.11449C > T	1.77	142.4	45.4	Yes	Yes
20	Male	WB	19	c.6526C > T	NK	1.63	77.3	28.9	Yes	Yes
21	Male	NK	18	c.2329C > T	c.2329C > T	1.63	100.9	38.2	Yes	Yes
22	Male	WB	49	c.10483C > T	c.10775delC	1.63	80	30	Yes	Yes
23	Male	SA	19	c.4937C > A	NK	1.47	62.1	28.7	Yes	Yes
24	Male	WB	36	c.10483C > Tter	c.9541C > Tter	1.62	84.9	33	No	Yes

*WB* White British, *SA* South Asian, *NK* not known

**Table 2:** Demographic, genetic and anthropometric characteristics of study participants (except who had neither kyphosis nor scoliosis)

ID	Sex	Ethnicity	Age (years)	Allele 1 nucleotide change	Allele 2 nucleotide change	Height (m)	Weight (Kg)	BMI (Kg/m^2^)	Deafness	Visual loss
25	Female	WB	21	c6584delA	c.8832C > G	1.63	72	26	Yes	partial
26	Male	WB	40	c.1729delA	c.10477C > T	1.72	109	37	Yes	Yes
27	Male	SA	5	c.5075delC	c.5075delC	1.07	31.6	27	Yes	partial
28	Male	WB	35	c.10769delC	c.10986G > A	1.65	82	30.1	Yes	Yes
29	Male	WB	17	c.10769delC	c.5356A > G	1.64	70	26	Yes	Yes
30	Female	WB	6	c.10769delC	c.10477C > T	1.02	23.35	22.4	NK	Yes
31	Female	WB	29	c.10769delC	NK	1.54	80.8	34	Yes	partial
32	Female	WB	32	c.10769delC	c.10986G > A	1.55	66	29	Yes	Yes
33	Female	SA	7	n.7678_10387chd	n.7678_10387chd	1.13	20.5	NK	No	Yes
34	Female	SA	7	c.4141delTCAC	c.4141delTCAC	1.17	36.35	26.5	Yes	No
35	Male	SA	12	c.8776C > T	c.8776C > T	1.37	45.5	24.2	Yes	Yes
36	Male	WB	42	c.8995C > T	c > 9001 C > T	1.6	109.7	42.8	Yes	Yes
37	Male	WB	25	c.6823C > T	c.9535C > T	1.64	69.4	25.8	NK	Yes
38	Female	WB	13	c.10477C > T	c.10769delC	1.33	34.6	19.6	Yes	partial
39	Female	WB	40	c.10477C > T	c.10477C > T	1.54	67.6	28.7	Yes	Yes
40	Female	SA	8	C.4937C > A	C.4937C > A	1.29	45	27	Yes	partial
41	Female	BA	19	c.2041C > T	NK	1.42	59.8	29.6	Yes	Yes
42	Male	WB	45	c.1874A > G	NK	1.8	104	32	Yes	Yes
43	Male	WB	25	NK	NK	1.7	71	24.6	Yes	Yes
44	Male	WB	20	NK	NK	1.66	92.4	33.4	Yes	partial
45	Male	WB	31	NK	NK	1.6	93.6	36.5	Yes	Yes
46	Male	WB	18	NK	NK	1.75	130.2	42.7	Yes	Yes
47	Male	SA	15	NK	NK	1.47	30.7	14.2	No	Yes
48	Female	WB	7	NK	NK	1.07	23.15	21	Yes	partial
49	Female	WB	7	NK	NK	1.04	17.5	NK	Yes	partial
50	Male	WB	4	NK	NK	0.93	20.15	23.4	No	partial
51	Male	SA	2	NK	NK	0.81	12.4	14.1	No	No
52	Male	NK	4	NK	NK	0.84	11.75	16.6	Yes	partial
53	Male	WB	41	NK	NK	NK	37.95	NK	NK	NK
54	Female	WB	51	NK	NK	1.44	62	29.9	NK	Yes
55	Male	NK	35	NK	NK	1.51	68.4	30	NK	Yes

*WB* White British, *SA* South Asian, *BA* British Asian, *NK* not known

Detailed examination at baseline and 12 months in 13 patients (ID 7-19 on Table 1 and supplementary Table 1) were performed. The summary of musculoskeletal deformities of these 13 cases, mean (range) aged of 25.3 (12–43), are shown in Table 3. Their mean height (range) and BMI was 1.58 (1.36–1.77) m and 30.3 (17.5–45.4) kg/m^2^, respectively. Their skeletal deformities did not change significantly between two points with 12-month interval.

**Table 3:** The summery of musculoskeletal manifestation in each region (n = 13)

Region	Musculoskeletal manifestations
Global	Short statue is not demonstrated until the early teens, Average height 157.5 cm
Cervical spine	3/13 had pain 12/13 had reduce movement, especially extension Lateral bend to the spine reported by carers. When interrogated in detail, it was to enable the ear with best audiological function to be well placed for function More than 50% had exaggerate forward curvature
Thoracic Spine	All had postural kyphosis—present as a correctable deformity in childhood. Postural advice allowed full correction No structural defects on radiographs None had a severe enough deformity to warrant surgical intervention or limit lung function
Lumbar spine	2/13 had scoliosis which required intervention 8/13 had scoliosis, but rarely noted by carers or patients, non-progressive and did not require intervention. These small curves are present in all ages. No structural defects on radiographs
Upper Limb	2/13 reported pain (Wrist and lateral humeral epicondylar ridge—tennis elbow), No functional deficits were found 5/13 had a fixed flexion at the elbow, did not affect function but did cause some difficulty with phlebotomy 2/13 had acromioclavicular tenderness on examination but did not report this as problematic in the questionnaire or on history taking 13/13 had Mild global brachydactyly
Hips	No incidence of congenital hip dislocation (CDH) or migration or significant dysplasia No symptoms were reported in relation to hips including trochanteric bursitis There was an internal rotation of the hips in prone position
Knees	No abnormality found on questionnaire or clinical examination No incidence of patello femoral pain or subluxation
Feet	11/13 had flexible pes planus 5/13 had notable tibialis posterior tenderness, none had dysfunction 2/13 reported medial ankle pain on questionnaire, but all said it was a feature everyday once examined, and history taken No other significant foot pathology No changes in keeping with severe diabetes such as neuropathy or skin ulceration

### Six cases of thoraco-lumbar scoliosis and case of spinal spondylitis

Surgical intervention was indicated in three patients with scoliosis (ID 1, 3, 5 on the Table 1 and supplementary Table 1) because of pain, and prognosis for deformity after adolescent growth.

**Fig. 1:**
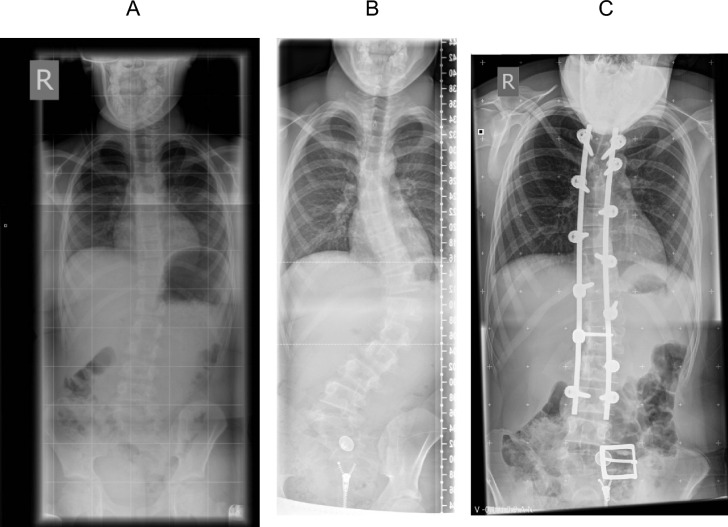
Case 1 (Case ID5); subject of this report. **A** PA whole spine radiograph at initial presentation demonstrates a mild scoliosis deformity. **B** PA whole spine radiograph demonstrating progression of the scoliosis. **C** Whole spine PA radiograph following surgical correction

Case 1 (ID 5): A 15-year-old male, presented to spinal unit at the age of 7 years. Initial radiographs revealed a mild left thoracolumbar scoliosis curve with a Cobb angle measurement of 10 degrees (Fig. 1A) which was managed with regular clinical and radiological surveillance. Between aged 13 and 14 in which his height increased; he reported an increase in clinical deformity. Clinical examination revealed coronal imbalance with waist and hip symmetry and normal neurological examination. Whole spine radiographs demonstrated a progression of the thoracolumbar curve, apex at T12, to a Cobb angle of 63 degrees (Fig. 1B) and his Risser grade was 0 (skeletally immature with significant growth potential remaining and hence the curve progressing further at a more rapid rate). The curve demonstrated good flexibility, reducing to 36 degrees on supine lateral bending films. MRI-spin revealed no abnormality of the neural axis.

In view of a large curve before skeletal maturity, and the anticipated progression during remaining growth and beyond into adulthood, surgery was recommended. A multidisciplinary team, including an anaesthetist, surgeon, cardiologist and paediatrician, involved in the preoperative planning and optimisation giving attention on his Alstom related complication in particular their dilated cardiomyopathy (DCM) which was stable (LVEF approximately 60%) and managed medically with Losartan, an angiotensin II receptor antagonist. A posterior instrumented correction and fusion from T5-L3 was performed. Multi-modal spinal cord neuro-monitoring was used. Pedicle screws were inserted along the concavity and convexity of the curve [11]. A dual rod correction was performed. Autogenous bone graft for fusion was harvested. No perioperative complications were noted and a satisfactory clinical and radiological postoperative follow up was completed to 18 months (Fig. 1C). Despite of experiencing in back ache on a regular basis, but rarely requiring painkillers after surgery, now he enjoy his daily activities including shopping and engaging with exercise at gym after 8 years of surgery. Obviously, there is a restriction in all trunk movements, particularly forward bend and rotation.

Case 2 (ID 1): A 36-year-old male had been treated surgically for his severe scoliosis at aged 14 because of pain. He had normal respiratory function and swam 20 lengths of a swimming pool three times a week.

Case 3 (ID 3): An 18-year-old female developed a significant scoliosis at age 13 years causing pain due to impingement of the chest wall on the iliac brim. This patient had two instrumented corrections at age 10, but she still has some upper thoracic scoliosis. Additionally bridging osteophytes is a noticeable feature in her vertebral X-ray (Supplementary Fig. 1).

Case 4 (ID 14): A 29-year-old female declined surgical correction for her severe scoliosis with impingement of the rib cage on the iliac crest resulting pain on walking and at night (Supplementary Fig. 2). Bridging osteophytes can be seen throughout the X-ray spine. Her pulse oximetry measurement during a six-minute walk remained > 90%.

Case 5 (ID 7) required regular physiotherapy to manage her symptoms.

Case 6 (ID 21): Severe clinical manifestations were reported requiring support with external brace.

Case 7 (ID 22): A 49-year-old male presented with neurological symptoms and MRI changes of diffuse idiopathic skeletal hyperostosis. He required successful decompression to relieve pain. He also developed similar changes around the hip joints which cause him to be a wheelchair user by 60 years of age (Supplementary Fig. 3).

## Discussion

Our large cohort study demonstrated the high prevalence of kyphosis and scoliosis among patients with Alström syndrome with confirming the well-known short stature demonstrated after early teens due to premature epiphyseal fusion and advanced bone age [4, 5, 13].

Kyphosis was the most prominent feature, detected in early childhood in all cases on clinical examination. Postural advice and exercises alleviated discomfort and improved appearance, with no patients requiring surgery or experiencing lung function impairment. Unlike the general adolescent population where kyphosis is rare, 40% of our participants exhibited kyphosis/stooping, possibly due to postural adaptations for dual sensory loss (hearing and vision), a hallmark of ALMS. Over 50% showed correctable exaggerated cervical forward curvature, which can lead to neck pain and reduced lung function by restricting lung volume and chest expansion if untreated. However, unlike kyphosis in ankylosing spondylitis in which there is a multilevel fused spine [14, 15], patients with ALSM had postural kyphosis. The high prevalence of kyphosis in this study was line with the phenotype description of the ALSM by Marshall et al. [5]. Early detection review for postural advice and exercises and physiotherapy are recommended, though surgery is rarely needed. Additionally, advanced phenoage in ALSM might, at least partly, contribute to the high prevalence of kyphosis.

Scoliosis, detected in adolescence, is the most severe clinical manifestation, with a few cases progressing rapidly to non-correctable deformities by postural advice requiring surgery, but most being subtle on clinical examination. These findings align with existing literature which stated the high prevalence of scoliosis and kyphosis in thoracic and lumbar regions [5, 12]. Lateral cervical spine bending, possibly to optimize auditory function, causes pain and reduced range of motion, particularly in extension. Mild scoliosis, especially in the lumbar region, is usually asymptomatic, non-progressive not having structural abnormalities on radiographs and rarely noted by caregivers or patients. Surgical correction, a treatment modality for the cases with severe deformity, pain, and hypoxia with pulmonary hypertension [16], was done for 3 patients for their extremely severe deformities. Despite structural defects on radiographs in all, none exhibited lung function limitations. The prevalence of scoliosis requiring intervention (10.7%) in our cohort is higher than the general population (surgery in adolescent idiopathic scoliosis is 0.01% of population). The characteristics of the scoliosis curves in our subject case were consistent with idiopathic rapid curve progression, likely accelerated by 18 years of higher phenotypic aging in individuals with ALMS1 mutation [17], requires careful monitoring. Notably, delayed puberty in ALMS male and precocious puberty in ALMS female need to be considered in anticipating curve progression timelines during surgical planning [18]. However, symptoms of some severe cases were managed with an external brace and ongoing physiotherapy. Only one AS patient exhibited severe spinal spondylitis which has been associated with severe insulin resistance in adults without diabetes [19, 20].

Based on our experience, managing spinal deformities in ALMS requires careful consideration of surgery risks due to comorbidities such as obesity, hyperlipidemia, insulin resistance, and organ fibrosis, which increase the risk of post-surgical organ failure [7–10]. The risk of perioperative complications is heightened by cardiomyopathy, which requires careful anaesthetic management [21, 22], particularly management of dilated cardiomyopathy (DCM) poses significant challenges, requiring careful selection of anaesthetic agents to avoid myocardial depression, maintenance of normovolaemia, and prevention of increased afterload [23]. Monitoring through central venous pressure and direct arterial pressure is essential, with inotropic support, if needed, and close postoperative care in a high-dependency unit. Visual dysfunction and sensorineural deafness should also be addressed to reduce distress and anxiety. Given the multi-system involvement in ALMS, a multidisciplinary approach is crucial for preoperative evaluation and optimization if surgery is deemed necessary. Successful surgical correction in three of our cases is encouraging. From the limited experience, instrumentation levels can be selected as for an idiopathic curve.

In addition, to spinal deformities, our study confirmed the well-known short stature in this cohort [4, 5, 13]. An increased tendency for other skeletal deformities, including brachydactyly and flexible pes planus, were other interesting findings in this large cohort for ultra rare disease. One of our patients had diffuse idiopathic skeletal hyperostosis requiring surgical intervention for symptom relief. Bridging osteophytes were noticed on vertebral X-rays in considerable number of the patient; unfortunately, we did not have exact statistic to give conclusion regarding the prevalence of hyperostosis in our study. However, our previous work has shown that in 35% of the patients with ALSM had very high bone mineral density that increases with age, in particular in the trabecular bones [24].Although none of participant in our population had congenital hip dislocation, 2 cases of late presenting hip dislocation have been found in the Alström national registry, which would give an incidence of CDH higher than expected. Interestingly, a wide range of the skeletal abnormalities in individuals with ALSM was reported in previous studies [5]. Additionally, individuals with ALMS may exhibit facial characteristics such as a round face, prominent forehead, and deep-set eyes and young looking for their age. Dental anomalies can include delayed tooth eruption, enamel hypoplasia, and malocclusion.

The role of *ALMS1* in skeletal deformities remains unexplored, but skeletal issues are common in ciliopathies [25, 26], suggesting ciliary protein complexes may influence cell polarity. We have recently shown an unusual increase in bone mineral density in patients with ALMS [24]. *ALMS1* regulates ciliary function and structure [3], mutations in this gene could contribute to the development of scoliosis in ALSM. Small lumbar spinal curves, present across ages, may reflect the disease process rather than provide a postural advantage. While the causes of idiopathic scoliosis are not fully understood, factors such as spinal muscle changes, collagen alterations, endocrine variations, spatial balance discrepancies, and genetic mutations have been suggested. In ALSM, broad endocrine and metabolic disturbances, along with obesity-induced mechanical stress, complicate this problem. Additionally, obesity might impose mechanical stress on spinal muscles, disrupting spatial balance. Notably, fibroblasts from ALMS patients produce excess extracellular matrix, including collagen. These findings highlight the need for further research on the role of the *ALMS1* gene in skeletal deformities.

ALMS patient fibroblasts also produce excess extracellular matrixes.

### This study has limitations

The ultra-rare nature of the syndrome led to small numbers of study subjects, but this is a large cohort of monogenic metabolic disease as a national service for patients with ALMS in the world. As a first study for skeletal deformities in patients with ALSM, there is limitation in data related to the quantitative measurement on the radiological abnormalities, thus further extensive study with systematic approach is warrant. Future studies will be required to investigate the causal link between the embryological and developmental effects of the ALSM mutation and scoliosis.

## Conclusions

Our study emphasizes the high prevalence of kyphosis, scoliosis, and other musculoskeletal changes in ALMS. In some with severe progressive scoliosis early detection and intervention are crucial to prevent severe deformities. Further research into the role of *ALMS1* in skeletal deformities is warranted.

## Supplementary Information

Supplementary file1

## Data Availability

Data are available on reasonable request.

## Abbreviations

ALMS: Alström syndrome
IR: Insulin resistance
NAFLD: Non-alcoholic fatty liver disease
PCOS: Polycystic ovarian syndrome
DCM: Dilated cardiomyopathy
