# Supplementary material for: Musculoskeletal deformities of Alström syndrome-a review of 55 cases
Source: Orphanet J Rare Dis. 2025 Jul 17;20:367. doi: 10.1186/s13023-025-03867-1 (PMC12272957; doi:10.1186/s13023-025-03867-1)

Supplementary table 1: Comorbidities of study participants with either kyphosis and/or scoliosis

| Case ID | spO2 on air | Smoker | LRTI | Asthma | Bronchiectasis | Type 2 DM | Blood pressure | Liver ultrasound | Creatinine | Cardiomyopathy | LVEF |
| --- | --- | --- | --- | --- | --- | --- | --- | --- | --- | --- | --- |
| 1 | 95+ | No | Yes | Yes | No | No | 100/46 | normal | 383 | No | 70 |
| 2 | 99 | No | No | No | No | Yes | 149/74 | normal | 82 | No | 85 |
| 3 | 97 | No | No | No | No | No | 141/93 | fatty liver | 50 | Yes | 63 |
| 4 | 95+ | No | No | No | No | No | 160/90 | normal | 70 | No | 55 |
| 5 | 95+ | No | No | Yes | No | Yes | 142/81 | normal | 81 | Yes | 68 |
| 6 | NK | No | Yes | Yes | Yes | Yes | 121/82 | fibrosis | 68 | Yes | 50 |
| 7 | 98 | No | No | Yes | No | No | 129/85 | normal | 45 | No | 63 |
| 8 | 89.4 | Yes | Yes | No | No | Yes | 136/70 | fatty liver | 415 | Yes | 41 |
| 9 | 95+ | No | Yes | No | No | Yes | 70/30 | cirrhotic | 316 | Yes | 30 |
| 10 | 95+ | No | No | No | No | Yes | 135/91 | normal | NK | Yes | 64 |
| 11 | 95+ | No | Yes | No | No | No | 190/100 | normal | 95 | Yes | 60 |
| 12 | 95+ | No | Yes | No | No | No | 109/59 | normal | 38 | Yes | - |
| 13 | 96 | No | No | No | No | Yes | 124/80 | normal | 435 | No | 60 |
| 14 | 95+ | No | No | No | No | Yes | 152/103 | normal | 62 | No | 77 |
| 15 | 95+ | No | No | No | No | No | 123/75 | fatty liver | 98 | No | 62 |
| 16 | 95+ | No | Yes | Yes | Yes | Yes | 121/82 | cirrhosis | 68 | Yes | 50 |
| 17 | 95+ | No | No | No | No | No | 122/79 | normal | 100 | Yes | 68 |
| 18 | 99 | No | Yes | Yes | No | No | 99/64 | normal | 35 | Yes | 63 |
| 19 | 95+ | No | No | No | No | No | 115/65 | fatty liver | 69 | No | 53 |
| 20 | 99 | No | No | No | No | Yes | 110/70 | fibrosis | 144 | No | 59 |
| 21 | 95+ | No | No | No | No | Yes | 120/78 | fatty liver | 82 | No | 57 |
| 22 | 96 | No | No | No | No | Yes | 134/83 | fibrosis | 70 | No | 60 |
| 23 | 98 | No | No | No | No | Yes | 124/56 | fatty liver | 78 | No | 67 |
| 24 | 95+ | No | No | No | No | No | 155/76 | fatty liver | 69 | Yes | 53 |

NK; not known

**Supplementary figure 1: Case 3 (Case ID 3)**


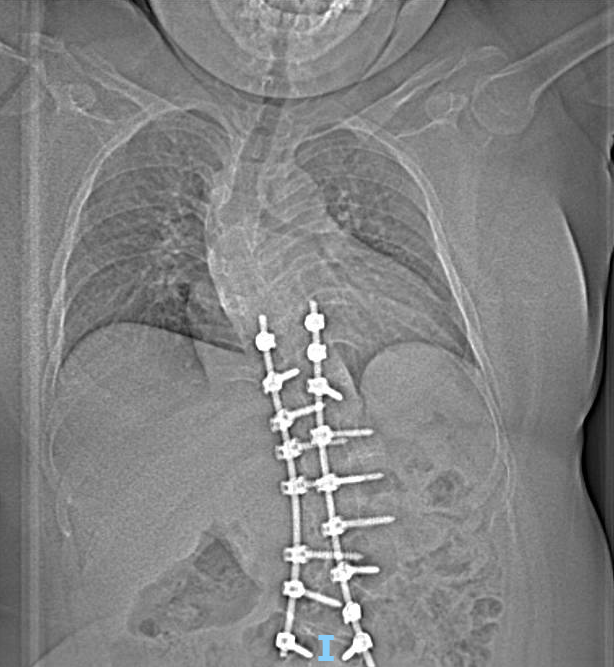


**Supplementary figure 2: case 4 (Case ID 14)**


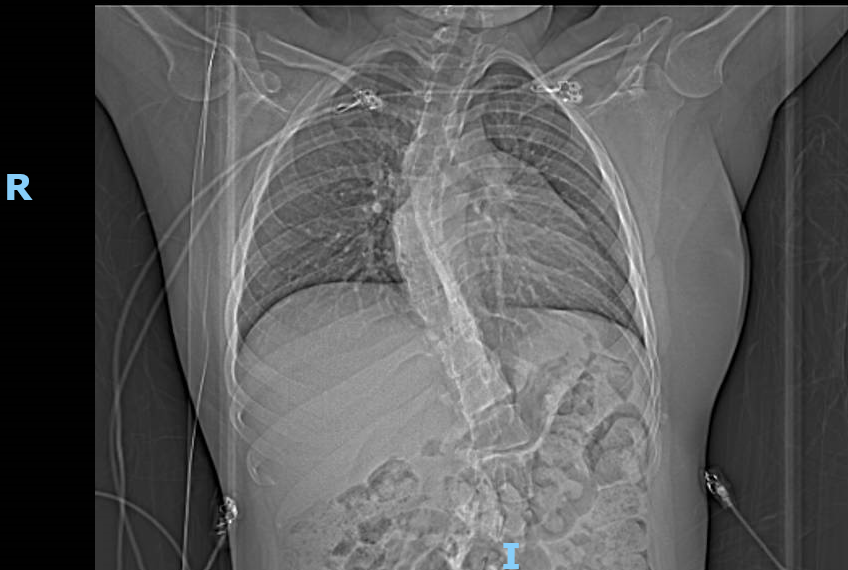


**Supplementary figure 3: case: case 7 (case ID 22)**

A: Xray neck B:X ray back


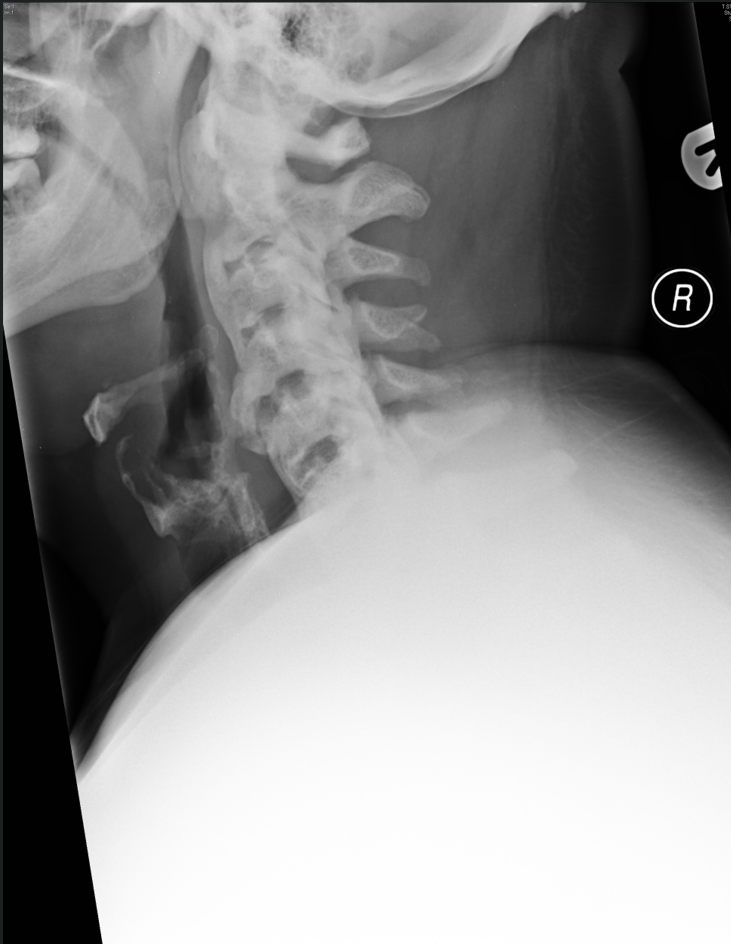

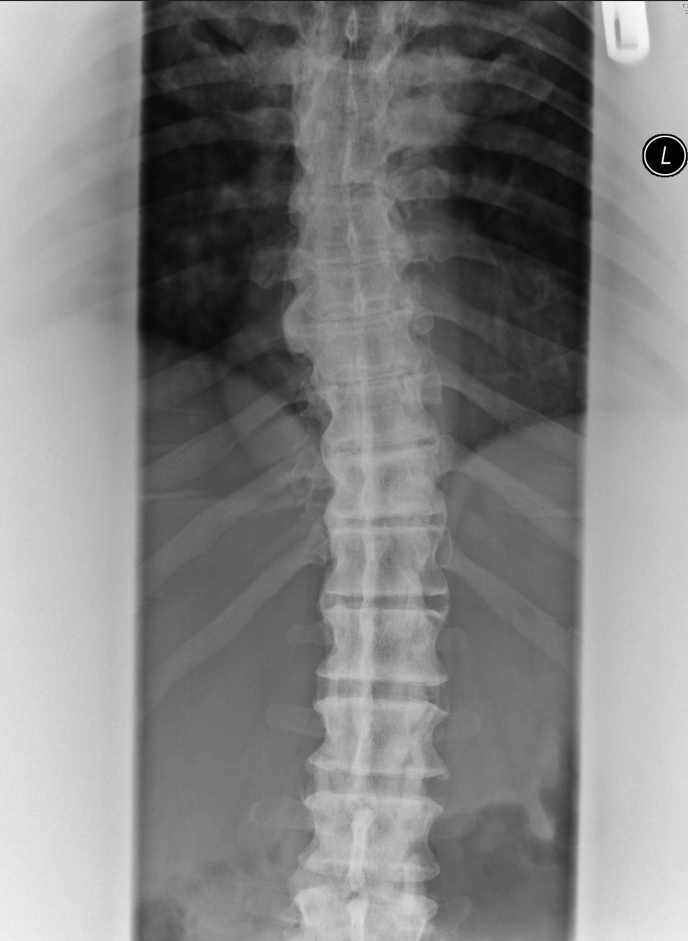

Supplement: Supplementary file 1 — Supplementary file1 [file 13023_2025_3867_MOESM1_ESM.docx]
